# Phage toxin variants are linked to protection specificity in a defensive symbiont

**DOI:** 10.1093/molbev/msag079

**Published:** 2026-03-24

**Authors:** Balig Panossian, Ailsa H C McLean, Vilas Patel, Taoping Wu, Muhammad Bilal Haider, Kerry M Oliver, Lee M Henry

**Affiliations:** School of Biological and Behavioural Sciences, Queen Mary University of London, London, UK; Department of Biology, University of Oxford, Oxford, UK; Department of Entomology, University of Georgia, Athens, GA, USA; School of Biological and Behavioural Sciences, Queen Mary University of London, London, UK; School of Biological and Behavioural Sciences, Queen Mary University of London, London, UK; Department of Entomology, University of Georgia, Athens, GA, USA; School of Biological and Behavioural Sciences, Queen Mary University of London, London, UK

**Keywords:** *Hamiltonella defensa*, symbiosis, host defense, parasitoid, genome evolution, bacteriophage

## Abstract

Insects often depend on symbiotic bacteria for protection; however, the mechanisms by which these microbes target specific natural enemies remain poorly understood. In aphids, different strains of the facultative symbiont *Hamiltonella defensa* provide highly specific protection against particular species of parasitoid wasps. To uncover the genetic basis of this specificity, we analyzed 26 *Hamiltonella* genomes and their toxin-encoding APSE bacteriophages with distinct protective phenotypes. Our analyses revealed that *Hamiltonella* strains share a conserved core genome but differ significantly in accessory gene content, reflecting their distinct evolutionary origins. Strikingly, we show that variation in toxin types is the key distinguishing feature of APSE phages in *Hamiltonella* strains that protect against different parasitoid species. These toxin repertoires include several novel candidates, such as variants with MAC/perforin domains and leucine-rich repeat (LRR) proteins previously unreported in insect defensive symbionts. We also reveal cases of multiple cointegrated APSE phages carrying different toxins within a single genomic locus. These findings suggest phage-borne toxins are important determinants of enemy-specific defense and point to phage-driven toxin diversification as a major force shaping the functional evolution of this symbiosis. This work highlights how mobile genetic elements influence the ecological roles and diversification of protective symbionts.

## Introduction

Insects are attacked by a myriad of natural enemies, driving the evolution of diverse defensive strategies. In addition to developing resistance through the host genome, many insects rely on symbiotic bacteria to defend against attackers (Oliver et al. 2014). Defensive symbioses have now been identified across the Insecta and involve partnerships with diverse microbial taxa that offer protection against a broad range of natural enemies (Cornwallis et al. 2023). This includes microbes that protect against sterilization from nematodes, infection by viruses, and attack from fungal pathogens and parasitoid wasps (Scarborough et al. 2005; Hedges et al. 2008; Vorburger et al. 2010; Yadav et al. 2018). Given the widespread occurrence of these beneficial associations and their substantial contribution to host protection, defensive symbioses are now recognized as important factors influencing insect-natural enemy population ecology and coevolutionary dynamics (Vorburger and Perlman 2018; Monticelli et al. 2019). Moreover, symbionts can provide highly targeted resistance against specific natural enemies (Kellner and Dettner 1996; McLean and Godfray 2015; Mateos et al. 2016); however, our understanding of the genetic mechanisms underlying this variation is limited.

One well-studied defensive symbiont is the gammaproteobacterium *Hamiltonella defensa*, hereafter *Hamiltonella*, which is estimated to occur in around 40% of aphid species and also psyllids and whiteflies (Clark et al. 1992; Russell et al. 2003; Henry et al. 2015). *Hamiltonella* is predominantly vertically transmitted but can also be horizontally transferred between host lineages and even species (Sandström et al. 2001). Studies have shown that *Hamiltonella* is typically nonrandomly distributed across aphid species and populations (Henry et al. 2022; Wu et al. 2022) and often occurs at high frequencies in host populations that carry the microbe. Although facultative, and therefore not essential for host survival, this symbiosis enhances host fitness by protecting against parasitoid wasps (Oliver et al. 2003). *Hamiltonella*'s defensive capabilities stem from its association with the bacteriophage APSE, which encodes eukaryotic toxins that target wasps (Van Der Wilk et al. 1999; Moran et al. 2005). Experimental evidence has confirmed APSE's role: phage loss eliminates protection (Oliver et al. 2009), while horizontal acquisition both in culture and in vivo restores the defensive phenotype (Brandt et al. 2017; Lynn-Bell et al. 2019). Homologous toxins in *Drosophila* also exhibit direct wasp-killing activity (Verster et al. 2023). Notably, *Hamiltonella* isolates vary in protection strength and specificity against different parasitoid wasp species (Oliver et al. 2005; Schmid et al. 2012; Cayetano and Vorburger 2013, 2015; Asplen et al. 2014; Martinez et al. 2014, 2016; McLean and Godfray 2015; Hopper et al. 2018; Wu et al. 2022). While specific toxin modules dictate the strength of protection against a single wasp species (Patel et al. 2023), the molecular basis of target specificity to different parasitoid species remains unclear.

The *Hamiltonella* strains found in European pea aphids (*Acyrthosiphon pisum*) provide striking examples of defensive variability (McLean and Godfray 2015; Leclair et al. 2016). In Europe, pea aphids form a complex of genetically differentiated, plant-associated biotypes (Peccoud et al. 2009), with populations on *Medicago sativa*, *Lotus pedunculatus*, and *Ononis spp*. (hereafter *Medicago*, *Lotus*, and *Ononis*) each predominantly harboring distinct *Hamiltonella* strains at high frequencies (Henry et al. 2013). McLean and Godfray (2015) found intriguing variation in the defensive properties of these strains—*Hamiltonella* from *Medicago-*associated pea aphids provided strong protection against the braconid parasitoid, *Aphidius ervi*, but not the chalcid parasitoid *Aphelinus abdominalis*, whereas *Hamiltonella* from *Lotus*-associated aphids protected against *A. abdominalis*, but not *A. ervi*. In contrast, the *Hamiltonella* from *Ononis-A. pisum* offered no protection against either of these natural enemies. A study by Wu et al (2022) found a similar pattern in that the *Hamiltonella* strains from *Macrosiphoniella artemisiae* and *Medicago* pea aphids each protected against their main parasitoid species (*Aphidius absinthii* and *A. ervi,* respectively), but not the other. Despite these striking differences in defensive capabilities, the genetic basis of this target specificity remains unknown. Here, we hypothesize that toxins encoded by APSE phage variants underlie parasitoid-specific defense.

In this study, we examined 26 *Hamiltonella* genomes, including 13 with known protective phenotypes, to explore the genomic basis of their defensive variation. This included 10 newly sequenced strains from the European pea aphid biotypes associated with *Medicago*, *Lotus*, and *Ononis* biotypes, two strains from *M. artemisiae* and *Macrosiphum euphorbiae* (Wu et al. 2022), and 16 publicly available genomes. Our genome analysis reveals that toxin modules are the key structural difference among APSE strains, with several novel toxins identified; our results suggest these variants underlie the strain-specific protection *Hamiltonella defensa* confers against different parasitoid wasps.

## Results

### Phylogenetic relationship among *Hamiltonella* lineages

**Figure 1:**
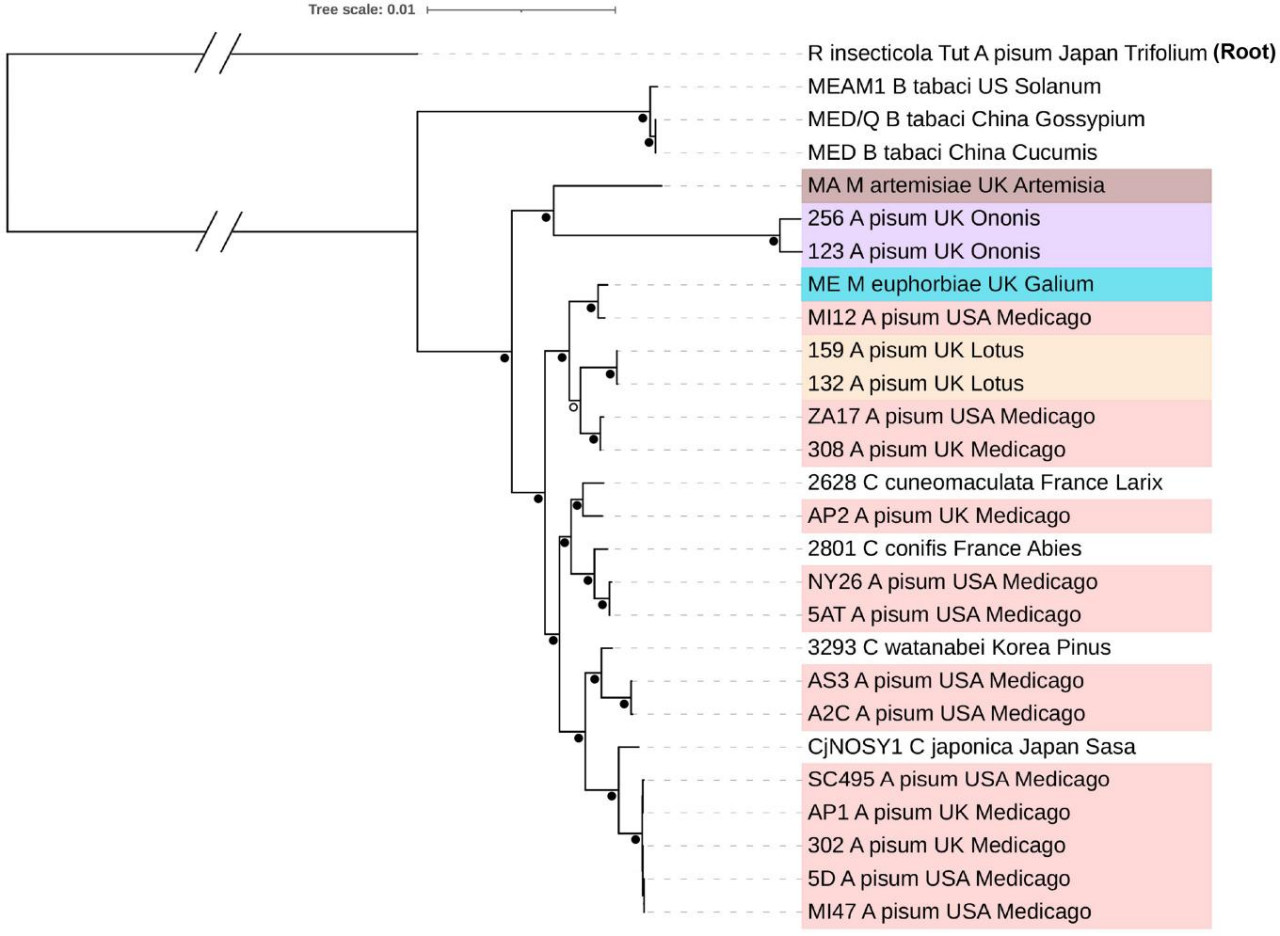
Maximum-likelihood phylogenetic tree of facultative *Hamiltonella* strains based on 147 housekeeping genes rooted to *R. insecticola*. Black circles (●) at nodes reflect high (>90) bootstrap support, and the open circle (○) reflects moderate (>70) support. Colors represent the plant association of each *Hamiltonella* strain; *M. artemisiae* in Brown, *M. euphorbia* in Light blue, and *A. pisum* biotypes *Ononis* in purple, *Medicago* in pink, and *Lotus* in orange.

We determined the phylogenetic placement of *Hamiltonella* strains included in our study by constructing a maximum likelihood (ML) phylogenetic tree based on 147 single-copy orthologous genes that included all published *Hamiltonella* genomes where it is a facultative symbiont. This produced a well-supported tree that notably places the *Hamiltonella* from the *Ononis* biotype of *A. pisum* and *Hamiltonella* from *M. artemisiae* as a distinct clade to other lineages sampled (Fig. 1). Although this placement is likely influenced by sampling limitations—most samples are from *A. pisum*—it demonstrates the *Hamiltonella* clade associated with *Ononis*-*A. pisum* and *M. artemisiae* form a distinct early branching sister lineage to other known lineages found in aphids. As shown in previous studies, we confirm that lineages from the *Medicago* biotype are paraphyletic (Henry et al. 2013; Guyomar et al. 2018) (Fig. 1). *Hamiltonella* from *Lotus* biotype *A. pisum* and *M. euphorbiae* aphids form a clade that includes several strains associated with the *Medicago* biotype (Fig. 1). Our results also confirm that *Medicago*-associated strains tend to form two major clades, with representatives from both found in the US and the UK. Interestingly, *Hamiltonella* lineages that belong to the largest clade associated with *A. pisum* on *Medicago* are closely related to several lineages collected from *Cinara* conifer aphids in France and Korea, as well as the social aphid *Ceratovacuna* in Japan. This demonstrates that *Hamiltonella* has been horizontally transferred across distantly related host species (Russell et al. 2003; Guyomar et al. 2018) and suggests horizontal transfer of *Hamiltonella* between pea aphid biotypes and other distantly related aphid species.

### Genome characteristics of aphid biotype- and species-associated *Hamiltonella* strains

We conducted detailed analyses on 19 *Hamiltonella* genomes from two sources. First, we sequenced 10 strains commonly associated with five aphid lineages in Europe—including *Lotus*, *Ononis*, and *Medicago* biotypes of the pea aphid (*A. pisum*), as well as the potato aphid (*M. euphorbiae*) and mugwort aphid (*M. artemisiae*), many of which have known protective phenotypes (Table 1). We then included nine previously sequenced *Hamiltonella* genomes from pea aphids collected on *Medicago* in the USA (Table 1, Table S1), some of which also have known defensive properties. We included *Hamiltonella* strains from *Cinara* and *Ceratovacuna* aphids and *Bemisia* whiteflies only in the phylogeny, given the limited experimental evidence for parasitoid-wasp protection. We found that *Hamiltonella* from *A. pisum* on *Ononis* (256, 123) have significantly smaller genomes, 1.845 ± 0.035 Mb, than strains from the *Medicago* biotype at 2.17 ± 0.032 Mb (Padj = 0.006**), and the *Hamiltonella* from *M. artemisiae* (MA) is also reduced in size at 1.94 Mb. The reduced genome sizes of the *Ononis* and MA *Hamiltonella* strains indicate substantial differences in genome content relative to other sampled lineages. We note that the strains from *Ononis* (256, 123) and *M. artemisiae* (MA) were sequenced with short-read technology, which could have an impact on their total genome sizes. However, sequencing coverage for these assemblies was fairly even, and highly covered short fragments only accounted for 3% to 5% variability of the total genome size, indicated it has a minimal impact on the observed genomes sizes. *Medicago*-associated *Hamiltonella* strains from Europe had largely similar genome characteristics to those from the USA (Table 1, Table S2). Phage and plasmid numbers were found to be variable across strains and biotypes. Although most genomes had >99% completeness and >100× coverage, we could not confirm the presence or absence of plasmids in all *Hamiltonella* strains (N/A's) due to sequencing with short read Illumina technology in some cases (Table S2).

**Table 1:** Metadata describing the Hamiltonella strains analyzed in this study.

Organism qualifier	Geographic location	Plant	Protective phenotype	Genome size (Mb)	Plasmids	Intact phages	Genome
5AT	USA	*Medicago*	Protection *A. ervi* (Moran et al. 2005)	2.169	1	2	Chevignon et al. (2018)
A2C	USA	*Medicago*	No Protection (phage lost) (Oliver et al. 2009)	2.204	3	2	Chevignon et al. (2018)
AS3	USA	*Medicago*	Protection *A. ervi* (Oliver et al. 2009)	2.262	2	3	Chevignon et al. (2018)
ZA17	USA	*Medicago*	N/A	2.273	3	1	Chevignon et al. (2018)
NY26	USA	*Medicago*	N/A	2.122	0	3	Chevignon et al. (2018)
MI47	USA	*Medicago*	N/A	2.276	5	3	Boyd et al. (2021)
5D	USA	*Medicago*	N/A	2.266	5	2	Boyd et al. (2021)
MI12	USA	*Medicago*	N/A	2.253	2	1	Boyd et al. (2021)
SC_495	USA	*Medicago*	N/A	1.949	N/A	1	Blow et al. (2020)
132	UK	*Lotus*	Protection *A. abdominalis*	2.08	4	4	This study
			No protection *A. ervi* (McLean et al. 2015)				
159	UK	*Lotus*	Protection *A. abdominalis*	2.05	3	3	This study
			No protection *A. ervi* (McLean et al. 2015)				
256	UK	*Ononis*	No protection (McLean et al. 2015)	1.81	N/A	2	This study
123	UK	*Ononis*	No Protection (McLean et al. 2015)	1.88	N/A	3	This study
308	UK	*Medicago*	Protection *A. ervi*	2.29	4	6	This study
			No protection *A. abdominalis* (McLean et al. 2015)				
302	UK	*Medicago*	Protection *A. ervi*	2.37	5	3	This study
			No protection *A. abdominalis* (McLean et al. 2015)				
MA	UK	*Artemisia*	Protection *A. absinthii*	1.94	N/A	2	This study
			No protection *A. ervi* (Wu et al. 2022)				
ME	UK	*Galium*	No protection *A. absinthii, A. rhopalosiphi, A. ervi* (Wu et al. 2022)	2.16	N/A	2	This study
AP1	UK	*Medicago*	Protection *A. rhopalosiphi, A. ervi* (Wu et al. 2022)	2.12	N/A	3	This study
AP2	UK	*Medicago*	No protection *A. rhopalosiphi*	2.03	N/A	4	This study
			Protection *A. ervi* (Wu et al. 2022)				

Protective phenotype describes the species of parasitoid wasp the *Hamiltonella* strain was tested against and whether it provided protection or not.

An analysis of gene synteny across long-read sequenced strains revealed substantial genome rearrangement across *Hamiltonella* lineages, with conserved synteny primarily restricted to closely related strains (Fig. S2).

### Gene content of European *Hamiltonella* strains differ in several functional categories

**Figure 2:**
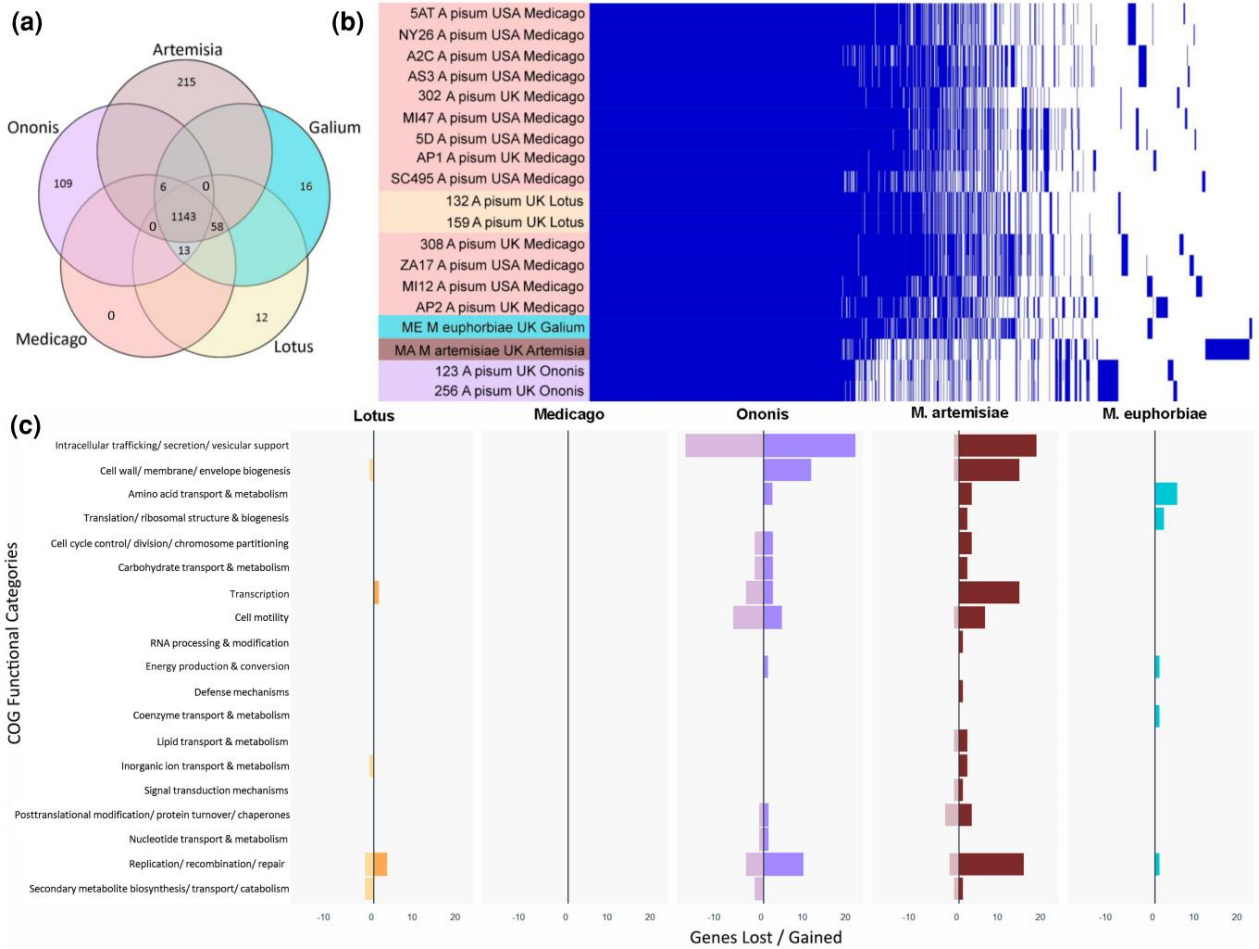
*Hamiltonella* genome comparisons. (a) Gene content distribution among the *Hamiltonella* genomes analyzed showing the number of shared/unique genes across the three European *A. pisum* biotypes (*Ononis*, *Medicago* and *Lotus*) and *M. euphorbiae* collected from *Galium aparine* and *M. artemisiae* from *Artemisia vulgaris*. b) Pangenome matrix of the *Hamiltonella* genomes illustrates the conserved core genome and highlights the unique accessory genome for samples of the five different biotypes. Colors represent *Hamiltonella* from *M. artemisiae* (brown), *M. euphorbiae* (light blue), and the *A. pisum Ononis* (purple), *Medicago* (pink), and *Lotus* (orange) biotypes. c) Functional categories of genes uniquely present/absent in the genomes of the ten new *Hamiltonella* genomes (two from each pea aphid biotype, one each from *M. euphorbiae* and *M. artemisiae*) from this study.

Our pangenome analysis of *Hamiltonella* revealed that strains share a large core genome but have notable differences in their accessory genomes (Fig. 2a, b). Only 12 and 16 genes were unique to *Hamiltonella* from each of the *A. pisum Lotus* biotype, and *M. euphorbia* from *Galium* sp., respectively, and no genes were uniquely present in the *Hamiltonella* from the *Medicago* biotype. In contrast, the *Ononis* biotype and *M. artemisiae* from *Artemisia* sp. associated *Hamiltonella* had the greatest gene content divergence, containing, respectively, 109 and 215 genes only found in these strains (Fig. 2a). All the genes uniquely present in the *Hamiltonella* from the *Lotus* biotype were APSE phage genes (Table S3). This highlights APSE variation as a key genomic feature associated with the contrasting defensive phenotypes.

A functional comparative analysis of European *Hamiltonella* genomes, based on Clusters of Orthologous Groups (COGs), revealed the greatest variability in three functional categories: (i) intracellular trafficking, secretion, and vesicular support (including secretion system machinery); (ii) replication, recombination, and repair; and (iii) cell wall/membrane/envelope biogenesis. These differences were particularly pronounced in *Hamiltonella* from the *A. pisum Ononis* biotype and from *M. artemisiae* (Fig. 2c).

Large differences in the number of genes involved in Intracellular trafficking, secretion system machinery, and vesicular support (COG category U; Table S3) were attributed to the *A. pisum Ononis Hamiltonella* losing genes that encode the Type II/IV secretion systems. Notably, *Ononis* strains were devoid of all 13 genes required to construct and assemble a functional Type 2 Secretion System (T2SS) as well as 18 genes required for the Type 4aP secretion system (T4aPSS) (Fig. S1). Loss of secretion systems, especially T2SS, may affect pathogenicity and within-host fitness in *Ononis Hamiltonella*, as these are key genetic factors underlying these traits in free-living *Yersinia* relatives (von Tils et al. 2012). In contrast, the genes gained by *Hamiltonella* from *M. artemisia* almost entirely coded for intracellular transporters (Table S3).

*Hamiltonella* strains also carried unique sets of genes involved in replication, recombination, and repair (COG category L; Table S3). These include bacteriophage mobilization genes, integrases, resolvases, and transposases. Genes uniquely missing from the *Ononis Hamiltonella* strains include a DNA repair gene *phrB* coding for a photolyase. Genes influencing cell wall, membrane, and envelope biogenesis (COG category M; Table S3) uniquely gained in the *Ononis Hamiltonella* include *eaeH*, which mediates attachment to eukaryotic cells (Sheikh et al. 2014), as well as genes controlling cell (*rfbF, rfbG, fcl*) (Zhang et al. 1993) and colony morphology (*ddhC*) (Anriany et al. 2006). *Ononis Hamiltonella* were uniquely missing the *licD* gene that alters the cell surface glycans to promote adherence and invasion. In contrast, *Hamiltonella* from *M. artemisia* uniquely carries the *licA*, *licC,* and *licD* genes, which are involved in eukaryotic mimicry and colonization (Zhang et al. 1999), and the *iabB* gene, which encodes for an invasion protein (Klein et al. 2000), and the adhesion gene *pmp10*. The *prgK* surface lipoprotein coding gene was also uniquely absent from the MA strain. *Hamiltonella* strains from the *A. pisum Medicago* and *Lotus* biotypes, and *M. euphorbiae,* did not show any unique gains and losses in genes coding for cell membrane structure. Together, these results suggest *Hamiltonella* strains differ in genes functionally important for interacting with hosts, which may contribute to host–symbiont incompatibility (Łukasik et al. 2015; Wu et al. 2022). Additionally, the *Hamiltonella* strains from *M. artemisiae* and *Ononis* pea aphids had a relatively higher number of cellular motility genes that were similar to bacterial flagella. Transcription factors were also detected at higher copy numbers in the *M. artemisiae* strain, some of which were multiple copies of core genes, which could be a result of duplication events. A full list of genes gained and lost, including a detailed annotation, can be found in the Supplementary material (Table S3).

Our genome analysis also revealed that the APSE phage was absent from the *Hamiltonella* ME strain from *M. euphorbiae* (Wu et al. 2022). As *Hamiltonella* can lose APSE upon transfer to laboratory colonies (Oliver et al. 2009; Lynn-Bell et al. 2019), this likely explains the ME strain's lack of protection reported previously (Wu et al. 2022).

### Variation in APSE phage toxin genes correlates with protective phenotypes

**Figure 3:**
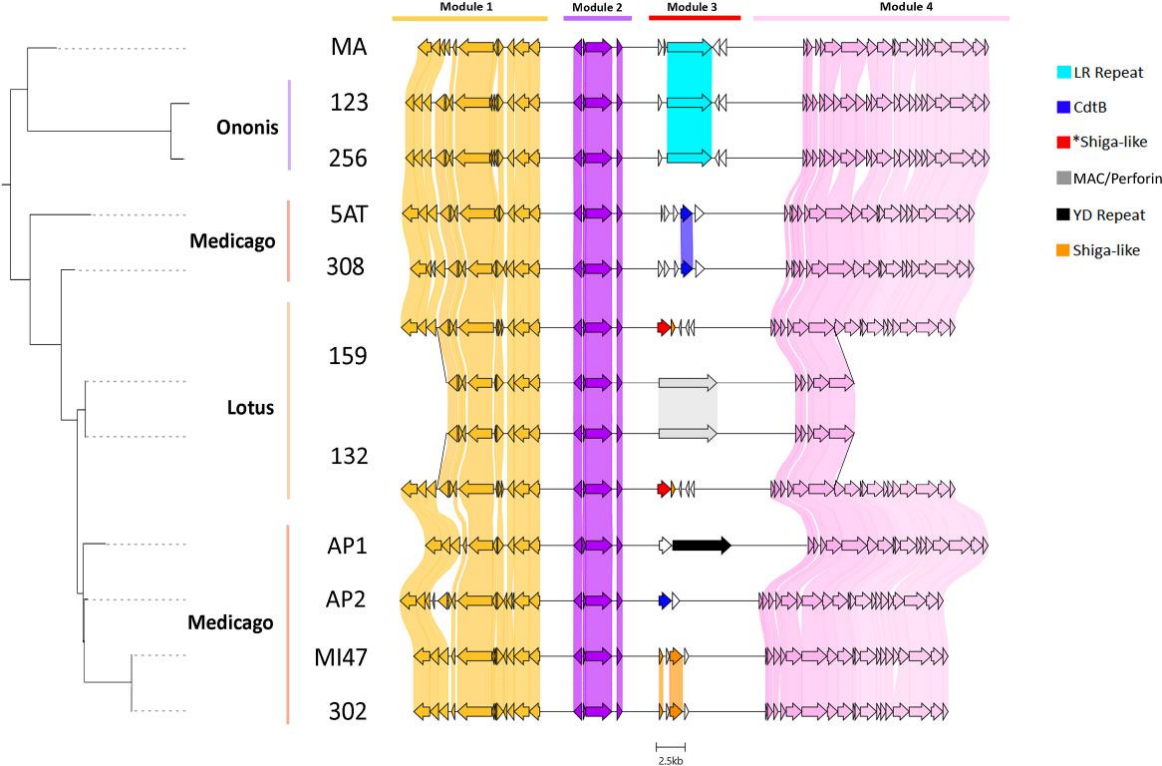
Comparison of APSE bacteriophage genomes from the newly sequenced European *Hamiltonella* genomes. Previously sequenced APSE strains 5AT and MI47 are included for comparison. Host plant (biotype) origins of each strain is indicated on the left and APSE modules are noted above. Note that most structural variation of the genomes occurs in Module 3, which contains genes encoding for eukaryotic toxins. In samples 132 and 159 from *Lotus* pea aphids, the genes of the two APSE phages are illustrated as two branches stemming from a shared APSE backbone, indicating the multiple copies of backbone genes and unique toxin-bearing modules integrated at the same genomic locus (Fig. S3). Phylogeny on the left is a pruned version of the conserved core APSE backbone from Figure S5, and modules are separated to improve readability. *Refers to the new Shiga-like toxin variant identified in this study (Fig. S4a).

We investigated the genetic basis of parasitoid-species–specific resistance in European *Hamiltonella* by comparing the ∼40-kb gene content of their associated APSE bacteriophages, key mediators of parasitoid defense. We found that while the replication and virion assembly modules of APSE phages are highly conserved, their toxin-encoding “module 3” exhibits striking diversity (Fig. 3), consistent with previous reports (Rouïl et al. 2020; Boyd et al. 2021).

Most notably, two *Hamiltonella* strains from the *Lotus* biotype (159 and 132)—which confer protection against the chalcid parasitoid *Aphelinus abdominalis*—harbor a unique APSE genotype consistent with multiple, cointegrated APSE phages within a single genomic locus (Fig. 3, Fig. S3). Each of these coresident phages carries a distinct toxin module. One encodes a novel variant of the previously characterized Shiga-like toxin (377 Amino Acids, AAs) in APSE-1 (Fig. S4a), but with the alpha and beta subunit genes arranged in reverse order and followed by three small transposases (Fig. 3). The second harbors a large (1678 AA) gene containing a membrane attack complex/perforin (MAC/Perforin) domain, marking the first report of such a toxin in *Hamiltonella* (Fig. 3, Fig. S4). Analysis of sequence data suggests these two APSEs are integrated within the exact same genetic locus in both strains (132, 159) of the two *Hamiltonella* genomes from *Lotus* biotype pea aphids. Although the precise orientation of the two toxin loci remains unresolved, haplotype-phased assembly graphs and read-level alignments suggest the two APSE phages share partial peripheral backbone Modules 1 and 4 and an identical, fully duplicated Module 2, after which each carries a distinct Module 3 encoding different toxin genes (Fig. 3, Fig. S3). Resolving the precise configuration of these toxin modules and determining how their combined presence influences resistance specificity will be an important focus of future work.

In *Hamiltonella* from the *Medicago* biotype, which protects against the braconid *A. ervi*, we found two toxin types: one phage encodes a cytolethal distending toxin B (*cdtB*; strain 308), while another (strain 302) carries a Shiga-like toxin distinct from that in the *Lotus* biotype. In contrast, the *Hamiltonella* strain from *M. artemisiae* (MA), which protects specifically against *A. absinthii* but not related *A. ervi* (Wu et al. 2022), contains an APSE phage with a large (1285 AA), highly divergent gene encoding a Leucine-Rich Repeat (LRR) domain protein (Fig. S4b). Similar LRR toxins were also found in APSE phages from the *Ononis* biotype (strains 123 and 256), with 97% nucleotide and 94% amino acid identity to the MA variant, suggesting that related APSE phages often encode similar toxin families (Fig. 3). A phylogenetic logistic mixed model allowing parasitoid-specific toxin effects was better supported than an additive model (ΔDIC = 20), consistent with toxin families being primary determinants of parasitoid-specific protection in this system (Table S4).

## Discussion

Our results show that variation in mobile genetic elements, specifically APSE bacteriophages and their toxin modules, is an important determinant of resistance specificity against different natural enemies. This suggests a dynamic interplay between *Hamiltonella* and different APSE phage isolates underlies variation in host protective phenotypes.

### APSE Phage toxin variation is associated with target-specific symbiont defense

Previous studies show that *Hamiltonella* often protects aphids specifically against the parasitoids that most frequently attack them in nature (Łukasik et al. 2015; McLean and Godfray 2015; Martinez et al. 2016; Wu et al. 2022). Our genomic analysis confirms that APSE phage backbones are highly conserved, whereas their toxin modules are hotspots of variation that correlate with parasitoid-specific protection (Boyd et al. 2021). We identified several novel toxin candidates, most notably a large protein containing a membrane attack complex/perforin (MAC/perforin) domain in the APSE phages of *Lotus*-associated *Hamiltonella*. This is the first report of such a toxin in *Hamiltonella* or its APSE phage. In other systems, including eukaryotic immunity and bacterial pathogenesis, MAC/perforin domain proteins function by oligomerizing to form pores in target cell membranes, leading to cell lysis (Dunstone and Tweten 2012).

These same APSEs, which protect against the chalcid *A. abdominalis*, also encode a divergent Shiga-like toxin, while other APSEs carry Shiga-like toxins that appear to target braconids such as *A. ervi* (Asplen et al. 2014; McLean and Godfray 2015). A highly conserved Shiga-toxin β subunit suggests similar binding to Gb3-like receptors, whereas divergence in the α subunit could alter ribosomal injury and thus host specificity (Melton-Celsa 2014). Resistance to *A. abdominalis* may therefore arise from the MAC/perforin toxin, the distinct Shiga-like variant, or their synergy. Regardless, these findings suggest that new toxin families and the sequence divergence of existing ones are potential evolutionary routes driving diversification of protective phenotypes in defensive symbioses.

Our genome comparison also revealed a LRR putative toxin gene in the APSEs of the *Hamiltonella* from *M. artemisiae* (MA) and *Ononis* pea-aphid biotypes. Despite having no known prokaryotic ortholog, its placement within the APSE toxin module, combined with the known virulence of LRR proteins in *Yersinia* (Hines et al. 2001), suggests a role in parasitoid defense. Consistent with high specificity, MA *Hamiltonella* protects *M. artemisiae* against the parasitoid *A. absinthii* but not *A. ervi* (Wu et al. 2022). Interestingly, the APSE phage in *Ononis Hamiltonella*, which is nearly identical to the one found in *M. artemisiae*, also does not protect against *A. ervi*, or *A. abdominalis* (McLean and Godfray 2015). This similarity suggests the toxin in the *Ononis* APSE may target an untested parasitoid. *Ononis* pea aphids are attacked by *A. aedyi* (Starý 2006), and parasitoid communities strongly predict *Hamiltonella* genotype distributions (Wu et al. 2025), and potentially, the APSE and toxins they carry. Future research should test whether the LRR gene found in *Ononis* APSE provides specific protection against *A. aedyi* and assess its activity against other parasitoids. Although the presence of previously undescribed toxin classes in APSE phages hints at novel mechanisms underlying parasitoid resistance, it should be noted that these require functional validation.

### APSE phage cointegration as a novel form of protection diversification

In *Lotus A. pisum* (clones 132 and 159), the APSE toxin module is duplicated, producing two module-3 copies that encode different toxins (Fig. S3). Our assemblies indicate these APSEs cointegrate at the same *Hamiltonella* locus—a rare genotype potentially formed by recombination at module boundaries (Rouïl et al. 2020). Although this is the first evidence of co-integrated phage occurring in *Hamiltonella*, analogous cointegration of Shiga-toxin carrying prophage does occur in *E. coli* (Nakamura et al. 2021). Such parallel cointegration could rapidly expand a symbiont's defensive arsenal by combining toxins rather than adding them sequentially. This highlights how modular, mobile phages may help generate multicomponent protective phenotypes in the host-parasitoid arms race. Thus, we have shown that APSEs in European *Hamiltonella* diversify not only via toxin-module variation but also through parallel co-integration within a single genome—a previously unrecognized route to enhanced defense.

## Conclusion

Mobile genetic elements (MGEs), particularly bacteriophages, are major engines of functional innovation in symbiotic bacteria (Hosokawa et al. 2016; Gerth et al. 2021; Siozios et al. 2024). Our work underscores the central role of MGEs, specifically bacteriophages, as drivers of functional innovation in defensive symbionts. We demonstrate that phage-encoded toxin repertoires are important determinants of target-specific protection in the *Hamiltonella*-aphid symbiosis, which evolve by both sequence divergence and the acquisition of novel families such as the MAC/perforin protein. We also show that phages can co-integrate within a single host genome, enabling the rapid assembly of multicomponent defenses. Together, these findings reveal how the modularity and mobility of phage genomes generate the variation and specificity needed for symbiont-mediated resistance, driving adaptation in the coevolutionary arms race between insects and their natural enemies.

## Materials and method

### Sample selection and genome sequencing

Ten aphid clones carrying *Hamiltonella* with distinct defensive phenotypes were included in this study (Table 1). Phenotypic data were collated from standardized laboratory challenge assays in three prior studies (Oliver et al. 2009; McLean and Godfray 2015; Wu et al. 2022). Across datasets, age-controlled aphid cohorts were exposed to single female parasitoids for fixed intervals, and protection was scored per individual as mummified (susceptible) or surviving (protected/resistant), excluding nonparasitoid mortality. Full assay conditions, including replicate numbers and exposure times, are detailed in the Supplementary material. Six pea aphid clones from *Lotus pedunculatus, Ononis spinosa, and Medicago sativa* were obtained from laboratory cultures at the University of Oxford (McLean and Godfray 2015). Potato aphid (*M. euphorbiae*), mugwort aphid (*M. artemisiae*), and two pea aphid lineages were clones maintained from a previous study in our lab (Wu et al. 2022). We attempted to in vitro culture all pea aphid *Hamiltonella* strains established through the methodology developed by Brandt et al (2017); however, only strains from *Lotus* and *Medicago* were successfully grown using this method. Sufficient concentrations of pure bacterial pellets were recovered, and DNA was extracted as described in (Weldon et al. 2013). Purified *Hamiltonella* genomes were sequenced on a PacBio Sequel II platform at an average yield of 88,851 × 9,300 bp reads per sample. Additional strains, including those that were unculturable, were sequenced using short-read Illumina technology. DNA was extracted from individual *Ononis, M. euphorbiae,* and *M. artemisiae* aphids using the EZNA Insect DNA kit (Omega Bio-Tek, Norcross, GA, USA), and they were sequenced on an Illumina NovaSeq S4 following the NEBNext Ultra II FS kit library prep protocol to generate paired-end reads of 2 × 150 bp length at an average of 200 million reads per sample. All samples were sequenced at the Centre for Genomic Research at the University of Liverpool.

### Genome assembly and quality control

Sequencing reads were checked for quality and adapter content using FastQC (Wingett and Andrew 2018) and MultiQC (Ewels et al. 2016) and were trimmed using FastP (Chen et al. 2018) with default parameters. Long reads were assembled using MetaFLYE v2.9 (Kolmogorov et al. 2020) with parameters –pacbio_hifi and 5 polishing iterations. Short reads were assembled in two steps. First, SPAdes v3.15.4 (Bankevich et al. 2012) was used in assembly only mode with default parameters. The reads were mapped to the assembly using BWA-mem (Li and Durbin 2009), and taxonomic identities were assigned using megablast (Morgulis et al. 2008) and DIAMOND (Buchfink et al. 2015) searches against the NCBI's nonredundant Refseq nucleotide and protein databases, respectively. These were examined using BlobTools v1.1.1 with default parameters (Laetsch and Blaxter 2017) where contigs matching *Hamiltonella* showing a distinct coverage and GC content were extracted (Fig. S6). Then, *Hamiltonella* reads were extracted using SAMtools (Li et al. 2009) and reassembled to give more contiguous and complete genomes with SPAdes v3.15.4 (Bankevich et al. 2012) in careful mode using error correction and kmer sizes of 33, 55, 77, 99, and 127.

All final assembly graphs were manually inspected in Bandage for uniform coverage and contiguity, and Blastn (Camacho et al. 2009) searches against the NCBI nonredundant nucleotide database were done to validate the taxonomic identities. Assembly completeness and contamination was assessed with CheckM (Parks et al. 2015) using f_*Enterobacteriaceae* as the marker lineage that has 546 genomic marker genes. Genomic markers and functional genes that were automatically assigned as missing were verified using HMM and blastp searches of the target genes on the unfiltered metagenome contigs.

### Phylogenetics

To determine the phylogenetic placement of the *Hamiltonella*s investigated in our study, we generated a phylogeny of all facultative *Hamiltonella*s with published genomes downloaded from GenBank. The GToTree v1.8.2 (Lee 2019) pipeline was run in default settings with 1,000 bootstraps in RAxML v8 (Stamatakis 2014) on a concatenated alignment of the 147 orthologous genes universally present in Gammaproteobacteria. The phylogeny was visualized and rooted to *Regiella insecticola* Tut (GCA_013373955.1) using ITOL (Letunic and Bork 2021). Root sensitivity was analyzed by rerunning the phylogeny with only *Hamiltonella* genomes (Fig. S7a) and again with *Fukatsuia symbiotica* as an outgroup (Fig. S7b).

Phylogenies of APSE phages were generated by identifying the core genes shared by all APSE phage backbones using Orthofinder v2.5.4 (Emms and Kelly 2019) run with default settings on the (.faa) amino acid sequence files of the APSE phages. Orthologs were aligned and concatenated using Muscle 5.1.linux64 (Edgar 2022) and TrimAL v1.4.rev15 (Capella-Gutiérrez et al. 2009). PhyML v3.0 (Guindon et al. 2010) was run with default settings and automatic model selection and 500 bootstraps to generate the phylogeny, which was visualized and rooted to the APSEs from *Hamiltonella* in *Bemisia tabaci* in ITOL (Letunic and Bork 2021).

To determine the phylogenetic placement of the newly identified shiga-like toxin alpha subunit, the Leucine-rich repeat, and MAC/Perforin domain containing toxin genes, we identified 100 of each of their closest hits through a BLASTp search on GenBank, which were aligned using Muscle 5.1.linux64 (Edgar 2022). The alignment was used in PhyML (Guindon et al. 2010) to generate a phylogenetic tree with default parameters and automatic model selection with 500 bootstraps. The tree was midpoint rooted, pruned, and visualized in ITOL (Letunic and Bork 2021).

### Annotation and pan-genome analysis

*Hamiltonella* genomes were annotated using Prokka v1.14.6 (Seemann 2014) with default settings in compliant mode. Genomes downloaded from GenBank were reannotated to ensure uniformity. Roary v3.13 (Page et al. 2015) was used with -e -i 90 -s settings to identify core genes common at (90% amino acid identity cutoff) in all genomes and accessory genes unique to *Hamiltonella* strains of each biotype. The pangenome matrix was visualized using the default Phandango web application (Hadfield et al. 2018). Genes uniquely present and absent in *Hamiltonella* from each biotype were annotated with COG, KEGG, GO terms, and Pfam domains using eggnog v6.0 via Eggnog-Mapper v2 run with -m diamond –itype proteins –sensmode sensitive –target_orthologs all and –go_evidence all (Hernández-Plaza et al. 2023). Visualization of the COG terms was done with GGplot2 in R v4.3.2 (Ihaka and Gentleman 1996). Genome synteny of long-read sequenced strains and references was calculated using ntSynt (Coombe et al. 2025a) with an estimated sequence divergence (-d) of 5 and was visualized using ntSynt-viz (Coombe et al. 2025b) using the pruned genome-based phylogenetic tree for –tree, a –scale of 500,000 basepairs, –seq_length 10,000, and –length 200,000.

### Comparative analysis of gene function

Pangenome analysis revealed major gene rearrangements belonged to three main functional groups—(i) intracellular trafficking, secretion, and vesicular support; (ii) replication, recombination, and repair; and (iii) cell wall/membrane/envelope biogenesis genes. After annotating the *Hamiltonella* genomes, we identified major functional groups of differences in gene presence and absence among lineages, with a particular focus on differences in functional categories of genes among genomes. We used Phaster (Arndt et al. 2016) to identify and extract bacteriophage sequences from the assemblies. It was run using default parameters, and only intact APSE regions were retained for comparative analyses of toxin content. Putative prophage regions were scored by PHASTER based on the proportion of phage-related genes and the presence of specific structural/functional phage components. Regions were classified as intact if they achieved a completeness score >90. The score derived by PHASTER was based on either high homology to known phages (providing a score up to 150) or by accumulating points for the presence of key phage features (e.g. +10 points each for capsid, tail, integrase, etc.; +10 for size >30 kb; +10 for >40 proteins). APSE phages from *Lotus* biotype *A. pisum Hamiltonella* strains were reassembled before comparative analyses, as explained in the section below. KEGG mapper (Kanehisa and Sato 2020) was used to check the presence of genes involved in metabolic pathways for amino acids, vitamins, carbon, lipids, and nucleotide metabolism, and transporter genes. The TXSscan HMM model was used through MacSyFinder v2 (Abby and Rocha 2017) using the unordered replicon parameter to search for genes coding for bacterial secretion system machinery. Genome assemblies were screened for plasmids by a BLASTn (Camacho et al. 2009) search using all plasmid sequences in GenBank previously identified in *Hamiltonella*, *Regiella*, and *Fukatsuia* genomes and those from our in vitro cultured long-read PacBio sequenced strains. All complete and incomplete phage and plasmid sequences were considered in the pangenome comparisons to avoid the loss of genes due to assembly fragmentation.

### APSE phage genome reconstruction

Bacteriophage contigs with annotated hits to APSE phages from the Phaster (Arndt et al. 2016) results were identified and flagged. *Hamiltonella* strains (132,159) from *Lotus* biotype *A. pisum* were reassembled using Hifiasm-meta v0.24.0-r702 (Feng et al. 2022) with the –primary parameter to resolve the dual haplotype modules 2 and 3 cointegrated into the same APSE backbone. In addition to both strains being in vitro cultured prior to sequencing to minimize environmental contamination, we conducted additional tests to validate APSE cointegration. CheckM indicated that there was no duplication of gammaproteobacterial single-copy marker genes, suggesting no mixed-strain assemblies. In addition, at the APSE integration locus, the chromosomal unitig is connected by two unambiguous long-read overlaps to toxin-bearing phage unitigs (Shiga-like and MAC/Perforin). These junctions were validated by mapping raw PacBio HiFi reads, with read-level alignments shown in Figure S3c–e (black lines in Fig. 3b). The initial assemblies were also checked for APSE sequences using Blastn searches of core APSE genes. The APSE genes were aligned and visualized using Clinker (Gilchrist and Chooi 2021).

### Statistics

Genome sizes were grouped by parent aphid species (and biotype for pea aphids) carrying each *Hamiltonella* strain. Standard errors were calculated for each group, and we used Levene's to test for variance homogeneity. ANOVA tested genome size differences among categories, followed by Tukey's HSD for post-hoc comparisons. Analyses were conducted in R v4.3.2 (Ihaka and Gentleman 1996) using dplyr, tidyr, and car packages.

To test whether APSE-encoded toxin families predict parasitoid-specific protection, we used a phylogenetic logistic mixed model in MCMCglmm (Hadfield 2010). Binary protection outcomes from individual host–parasitoid assays were analyzed with *Hamiltonella* strain fitted as a phylogenetic random effect. An additive model including parasitoid identity and toxin presence/absence was compared to a model allowing parasitoid-specific toxin effects via parasitoid × toxin interactions. Models used a categorical (probit) error structure, and support for specificity was assessed using ΔDIC, with effect sizes reported as posterior means and 95% credible intervals.

## Supplementary Material

msag079_Supplementary_Data

## Data Availability

The genome assemblies generated in this study have been deposited in the NCBI GenBank database under BioProject accession number PRJNA1338369.
