# Supplementary material for: Phage toxin variants are linked to protection specificity in a defensive symbiont
Source: Mol Biol Evol. 2026 Mar 24;43(5):msag079. doi: 10.1093/molbev/msag079 (PMC13137986; doi:10.1093/molbev/msag079)
Supplement: msag079_Supplementary_Data [file msag079_supplementary_data.zip › Toxin paper supplementary figures FEB2026_Cleaned.docx]

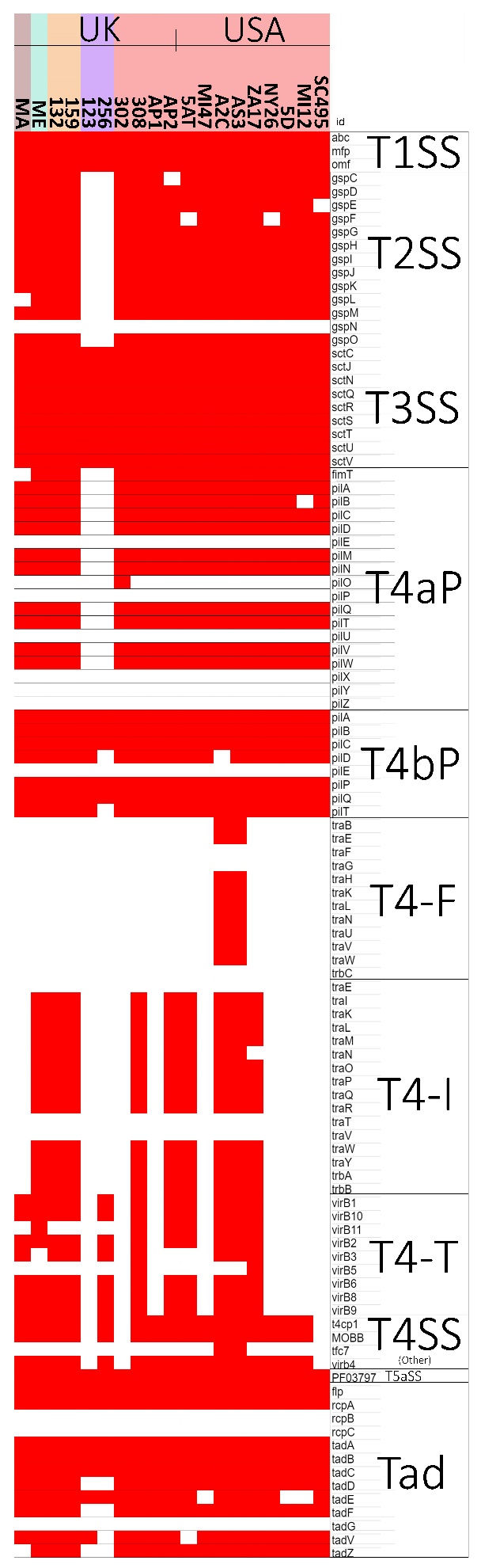


**Figure S1**. Matrix showing the presence (red) and absence (white) of genes involved in forming the secretion system machinery in *H. defensa* genomes across five biotypes from the UK and the US.


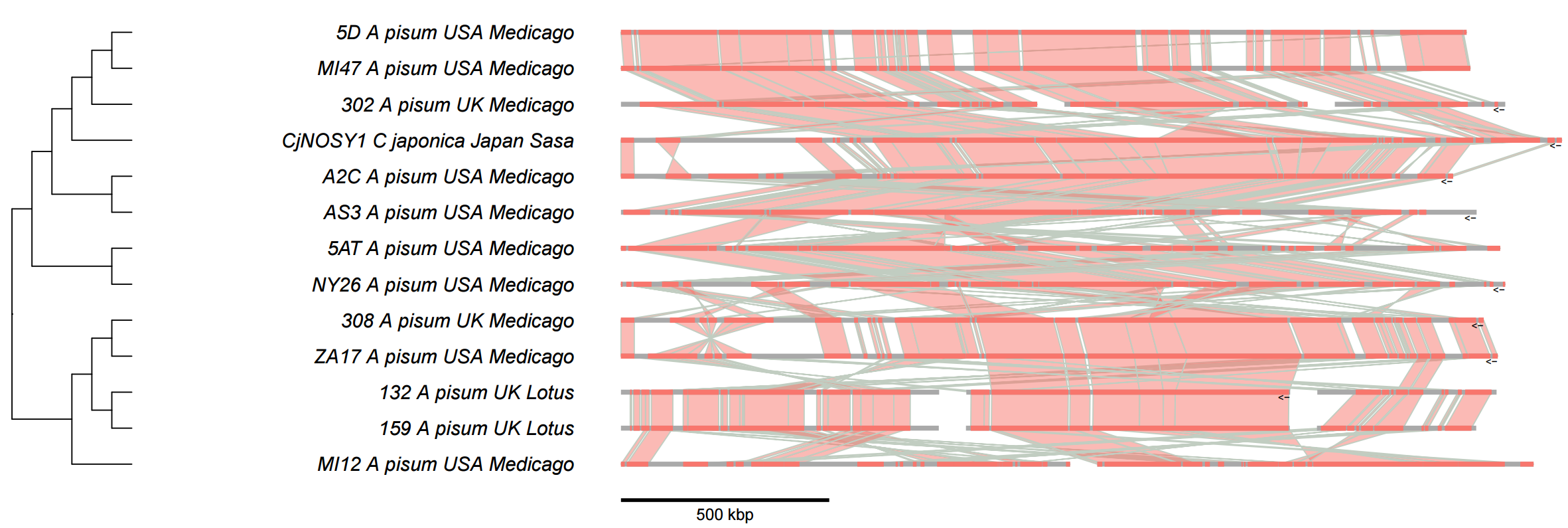


**Figure S2**. Gene synteny of long-read sequenced strains ordered according to the pruned genome phylogeny (Fig. 3). Pink lines represent 10Kb syntentic fragments where as those in grey represent those between 3-10Kb. size.

**Figure S3.** Technical evidence suggesting the presence of multiple APSE strains in the Lotus *Hamiltonella* samples ( 132 and 159 ).

A ) Coverage graph showing the number of sequencing reads successfully mapping to one reference APSE sequence. The graph shows a 300x coverage of the initial few APSE genes of module 1, at a similar coverage to the *Hamiltonella defensa* genome which was also sequenced at 300x coverage. The coverage then increases to 600x, suggesting two copies of the last few genes of module 1. The coverage of module 2 is at 600x throughout, suggesting a fully duplicated module 2. A sudden drop from 600x to 300x is seen at the border of toxin-bearing module 3, indicating that each half the APSE reads correspond to each of the two identified toxins. The results are identical when the reference APSE is swapped to the one carrying the other toxin gene, giving each module 3 a 300x coverage from the reads generated from sequencing the same initial *Hamiltonella* sample. Coverage increases to above 300x after the toxin module, suggesting that in module 4, each APSE strain has its own few unique genes initially, whereas the last genes of module 4 seem to be shared due to the coverage drop of back to 300x.

B) Assembly graph of the haplotype-phased unitigs in the step prior to the complete assembly of the genome sequence. From left to right, the graph shows the contiguous *Hamiltonella defensa* genome which cannot be phased into different haplotypes until reaching the APSE locus. The first 5 genes of module 1 are shared, followed by 2 haplotypes of the APSE backbone, each carrying one of the two identified toxin genes, after which the graph converges back into one haplotype carrying most of the APSE backbone genes in module 4. Three junctions spanning the edge-connected unitigs are annotated with dashed arcs labeled as J.1-3 showing assembler-detected long-read sequence overlaps represented by the black lines.

C-E) Long read alignments to the annotated junctions connecting the APSE backbone to both toxin-carrying modules each with a partial share of the total reads. Colours represent forward and reverse reads, demonstrating reads in both directions span each junction.


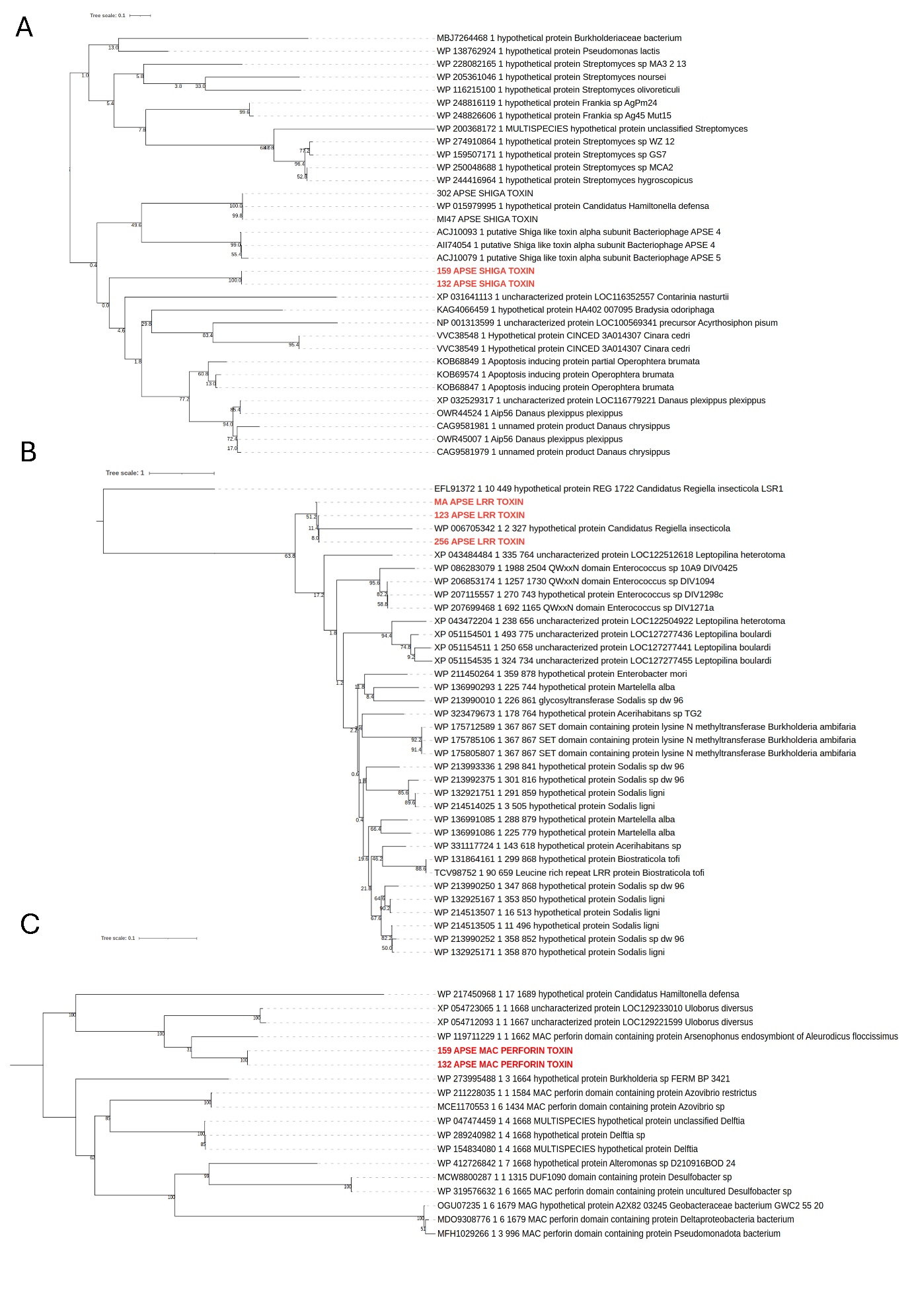


**Figure S4**. Maximum likelihood amino acid phylogenies of the newly identified APSE toxin genes highlighted in red text. Trees were rooted at the midpoint to allow for the phylogenetic placement of each toxin gene among closest related protein coding sequences on Genbank. A) Phylogeny of a new shiga-like toxin gene found in the APSE bacteriophages of *Hamiltonella* from *Lotus* biotype (strains 132,159) pea aphids. The gene is shown to be adjacent to a clade of previously identified Shiga-like toxins from APSE phages. B) Phylogeny of a new Leucine-rich-repeat domain containing toxin gene found in the APSE bacteriophages of *Hamiltonella* from *Ononis* biotype (strains 123, 256) pea aphids and *Hamiltonella* (strain MA) from the *M. artemisiae* aphid. Low bootstrap values imply the lack of closely related genes. C) Phylogeny of a new MAC/Perforin domain containing putative toxin found in the APSE bacteriophages of *Hamiltonella* from *Lotus* biotype (strains 132, 159).

**
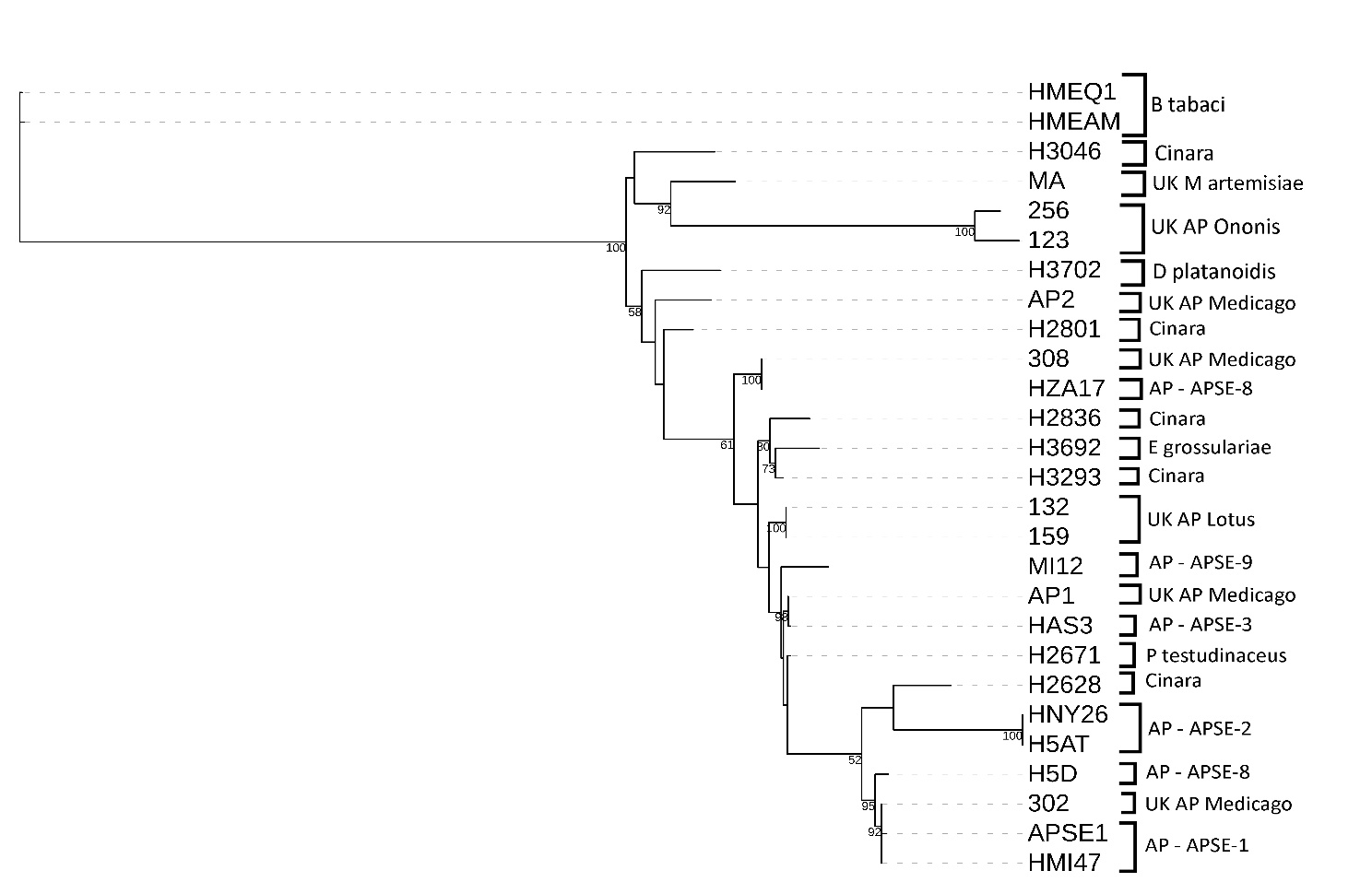
**

**Figure S5**. Maximum-Likelihood core gene phylogeny of the orthologous genes found in all APSE backbones built with 500 bootstraps. Tips of phylogeny are annotated with original sample names of their respective *Hamiltonella defensa* host genomic sources, followed by the parent insect names in brackets.

**
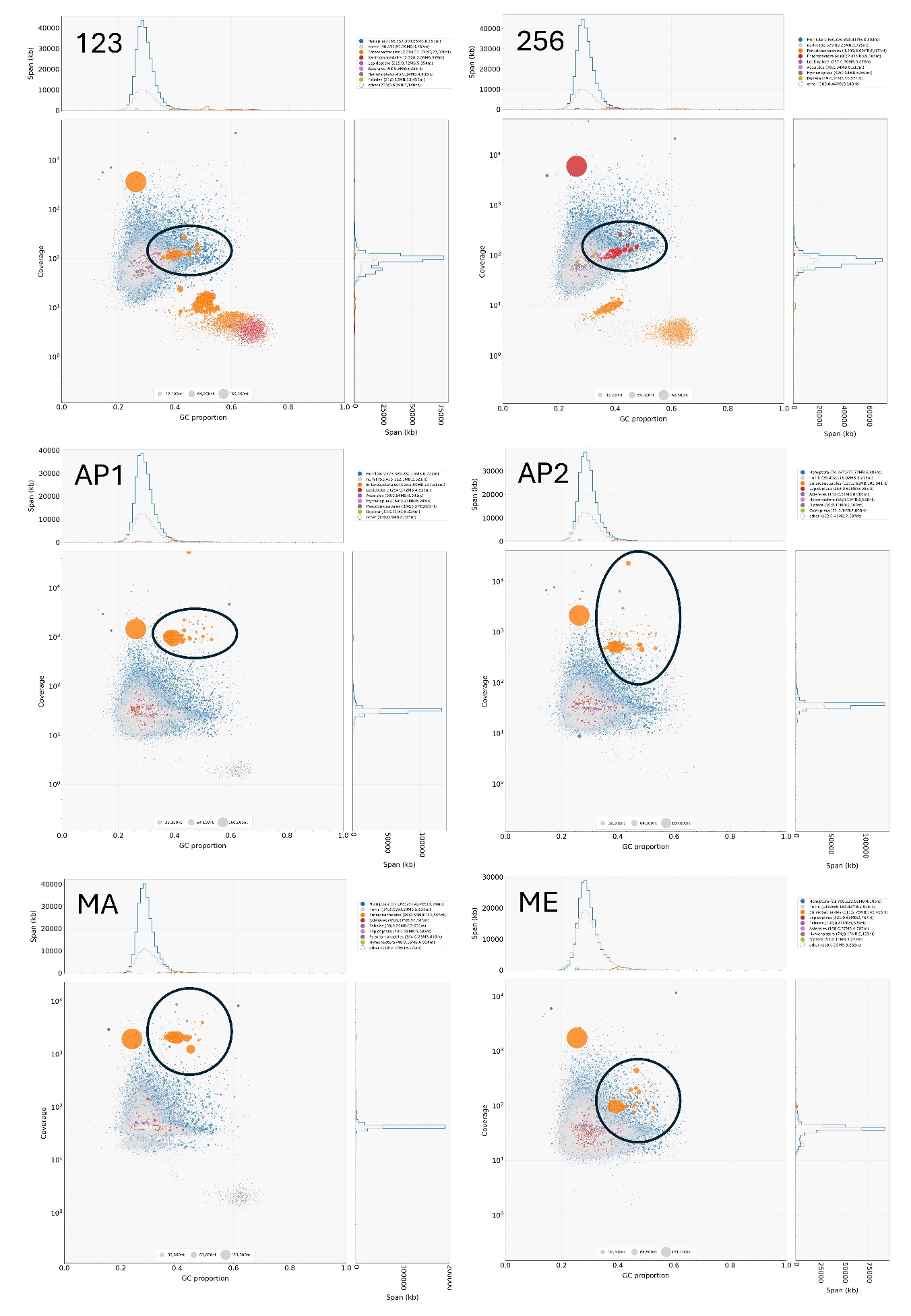
Figure S6**. Blobplots of the Illumina sequenced samples. Initial assemblies of entire metagenomes showing the distribution of sequence coverage on the Y axis, GC% on the X axis, taxonomic ID through different colours of blobs, and sizes of initial contigs by the size of the blobs. Blobs corresponding to *Hamiltonella* genomes are encircled.

A.


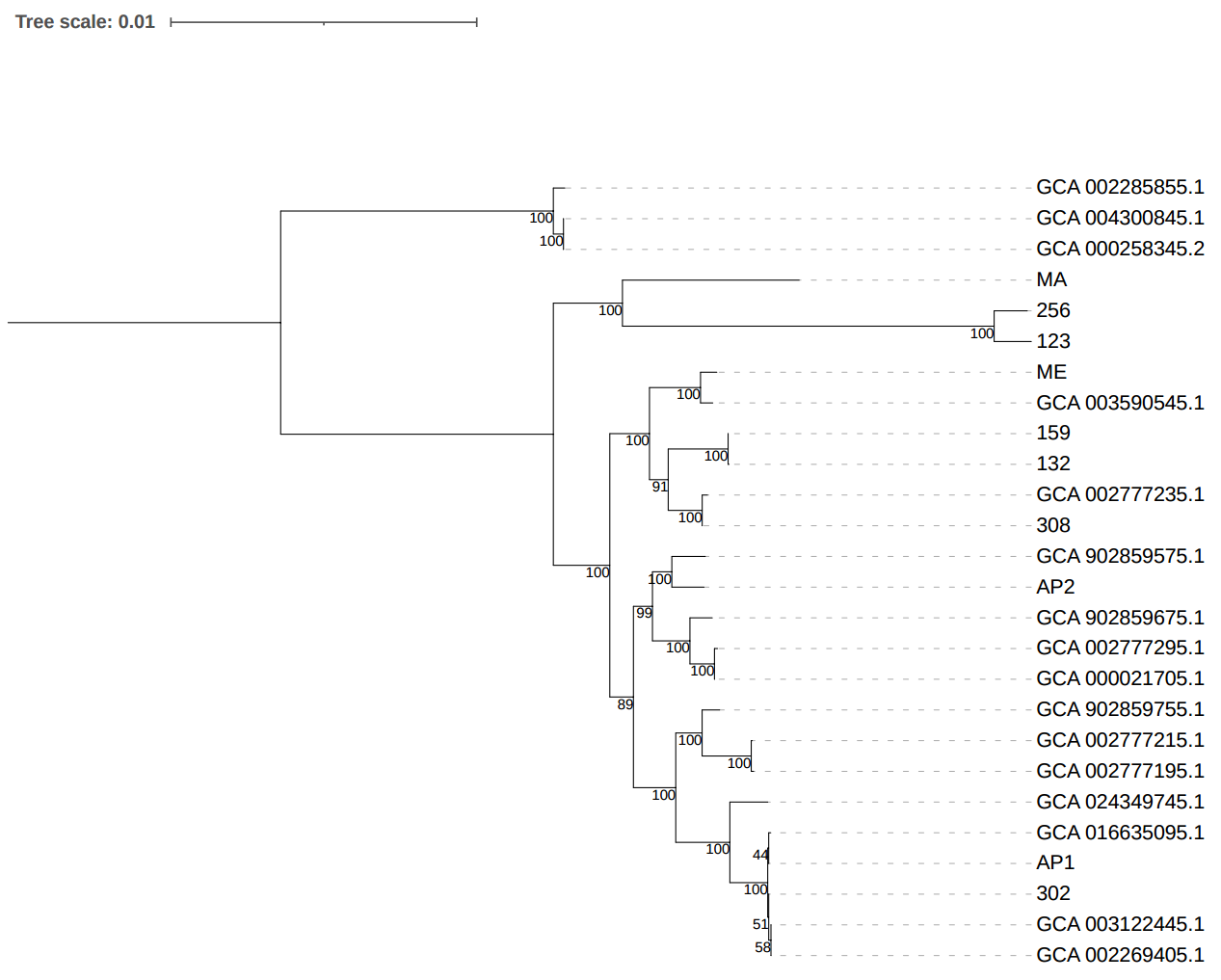


B.

*
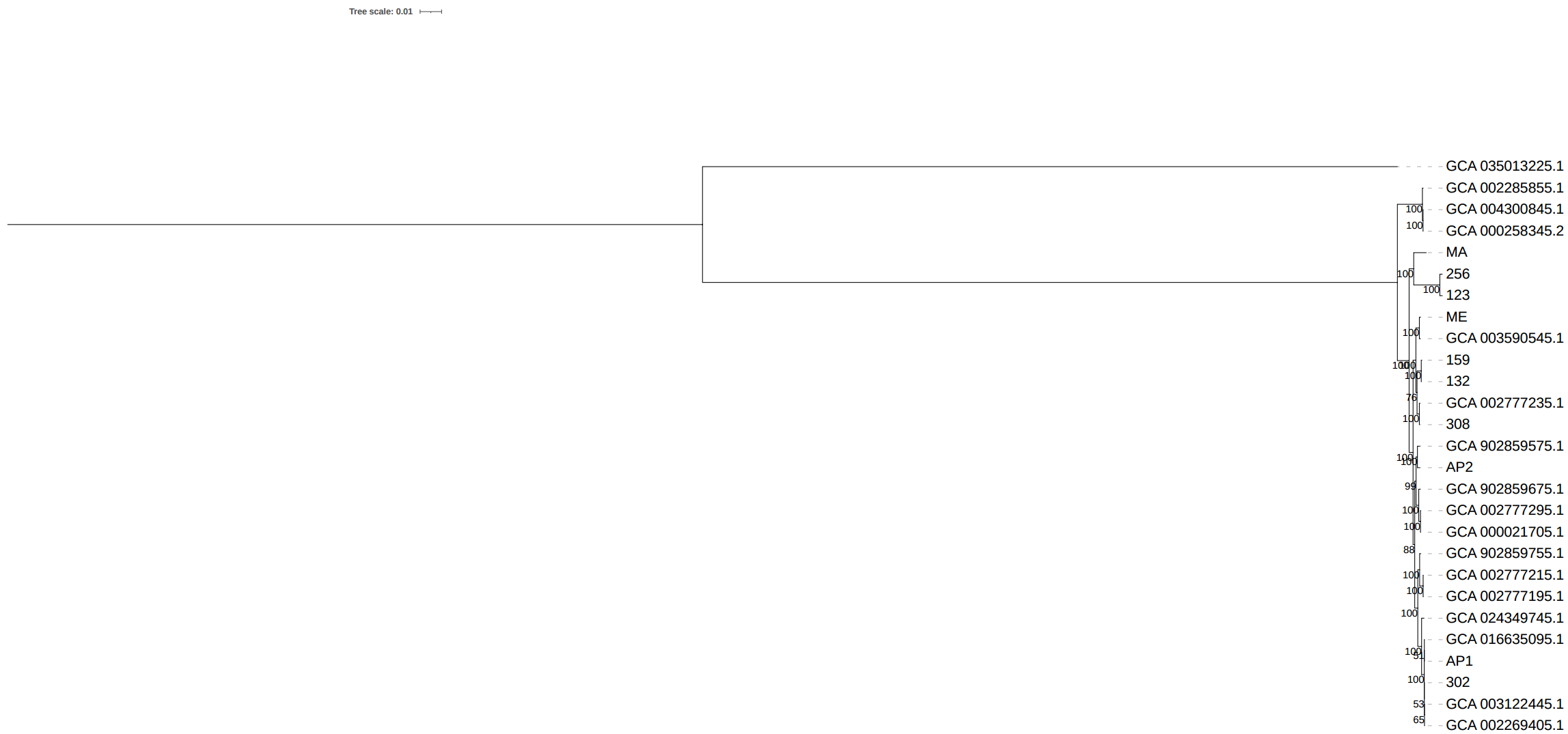
*

Figure S7. Maximum-Likelihood phylogenies of facultative *Hamiltonella* strains based on 147 housekeeping genes rooted to A) *Hamiltonella* strains from *B. tabaci* B) *Fukatsuia* *symbiotica.*

*Conserved genomic architecture of Hamiltonella enables the identification of key evolutionary changes*

The *Hamiltonella* genomes studied were largely conserved in terms of phylogenetic relatedness and the number of shared core genes. This limited divergence enabled us to compare the evolutionary differences in functional gene gains and losses that are unique to strains that are typically found in different aphid hosts. *Hamiltonella* strains from *Ononis* pea aphids and *M. artemisiae* were the most phylogenetically basal strains, as well as the ones with the smallest genomes. This suggests that the large differences in genome content between these strains and the other genomes of *Hamiltonella* is due to them being more ancestral relative to the others, suggesting evolutionary divergence and possibly genomic erosion over a longer period of time (McCutcheon & Moran, 2012; Rispe & Moran, 2000). The ancestral genomes contain similar APSE phages that carry the LRR-domain-containing toxin, indicating their descent from a common ancestor. The absence of Type 2 and 4 secretion system machinery in the *Ononis* strains of *Hamiltonella,* in particular, the genes coding for the Type 2 secretion system are regarded as important genetic factors that influenced the pathogenicity and environmental fitness of *Yersinia* species that are its closest free living relatives (Degnan et al., 2009; von Tils et al., 2012). Moreover, the large divergence in functional gene content in the strains indicate a history of divergent evolution from the more recently diversified strains of *Hamiltonella*. The basal strains of *Hamiltonella* from the UK give us an insight on how different evolutionary routes may lead to these specialised protective genotypes through gains, in shared functional categories, of genes having slightly different mechanisms and cellular targets (Boyd et al., 2021; Rouïl et al., 2020).

Our findings highlight the diverse evolutionary trajectories of previously unexplored *Hamiltonella* strains from *Ononis* pea aphids and *M. artemisia* aphids. *Ononis* *Hamiltonella* appear to have uniquely gained, or are the only ones to have retained, genes modulating eukaryote host colonisation such as eaeH (Sheikh et al., 2014), as well as cell (rfbF/G, fcl) (Zhang et al., 1993) and colony morphology (ddhC) (Anriany et al., 2006). On the other hand, *Hamiltonella* strain MA from *M. artemisia* may engage with eukaryotes through mimicry by carrying genes (LicA LicC and LicD) (Zhang et al., 1999) and invasion through the (iagB) gene (Klein et al., 2000).

No genes were observed to be fully unique to Medicago *Hamiltonella* strains, and the phylogeny showed that Medicago strains are not monophyletic in both pea aphids from the US and the UK. The presence of *Hamiltonella* genomes from conifer and bamboo aphids between the different clades of Medicago *Hamiltonella* could suggest a horizontal acquisition of *Hamiltonella* between aphids on these plants (Henry et al., 2013).

*Phenotypic assay conditions*

Protective phenotypes were derived from controlled parasitism assays where *Hamiltonella*-infected and cured aphid lines were exposed to parasitoid wasps. While specific parameters varied slightly by study, all assays followed a uniform design principle allowing for the binary coding of individual aphids as either protected (surviving) or susceptible (mummified).

Oliver et al. (2009):

- Host system: Cohorts of 20 second-instar *A. pisum* numphs were utilised per replicate.

- Parasitoid challenge: Each cohort was exposed to a single *A. ervi* female in a 6cm Petri dish. Parasitised aphids were subsequently transferred to potted *V. faba* plants and reared at 20°C (16L:8D).

- Scoring: Susceptibility was determined by counting the number of surviving versus mummified aphids.

Mclean & Godfrey (2015):

- Host system: Cohorts of 15 third-instar *A. pisum* nymphs (4-5 days old) were utilised per replicate.

- Parasitoid challenge: Each cohort was exposed to a single female of either *A. abdominalis* or *A. ervi*. Exposure duration was fixed at 24h for *A. abdominalis* and 3h for *A. ervi* to minimize superparasitism.

- Scoring: Susceptibility was determined by recording mummification rates at 10 days (*Aphidius*) or 14 days (*Aphelinus*) post-exposure.

Wu et al. (2022):

- Host system: Cohorts of 15 second-instar nymphs ( of *M. artemisiae*, *A. pisum,* or *M. euphorbiae*) were utilised per replicate (n ≥ 12 replicates per line).

- Parasitoid challenge: Each cohort was exposed to a single female of the corresponding parasitoid species (*A. absinthii, A. ervi,* or *A. rhopalosiphi*, respectively) for a fixed duration of 24 hours.

- Scoring: Susceptibility was determined by calculating the mummification rate of the cohort 12 days post-exposure.
